# Germline BRCA 1-2 status prediction through ovarian ultrasound images radiogenomics: a hypothesis generating study (PROBE study)

**DOI:** 10.1038/s41598-020-73505-2

**Published:** 2020-10-05

**Authors:** Camilla Nero, Francesca Ciccarone, Luca Boldrini, Jacopo Lenkowicz, Ida Paris, Ettore Domenico Capoluongo, Antonia Carla Testa, Anna Fagotti, Vincenzo Valentini, Giovanni Scambia

**Affiliations:** 1 Dipartimento per le Scienze della salute della donna, del bambino e di sanità pubblica, Fondazione Policlinico Universitario A. Gemelli IRCCS, Gynecologic Oncology, Rome, Italy; 2 Dipartimento di Diagnostica per immagini, radioterapia oncologica ed ematologia, Fondazione Policlinico Universitario A. Gemelli IRCCS, Rome, Italy; 3 Department of Molecular Medicine and Medical Biotechnology, Federico II University-CEINGE, Advanced Biotechnology, Naples, Italy; 4 Department of Obstetrics and Gynecology, Fondazione Policlinico Universitario Agostino Gemelli IRCCS, Catholic University of the Sacred Heart, L.go A. Gemelli 8, 00168 Rome, Italy

**Keywords:** Medical genetics, Cancer genetics, Cancer prevention

## Abstract

Radiogenomics is a specific application of radiomics where imaging features are linked to genomic profiles. We aim to develop a radiogenomics model based on ovarian US images for predicting germline *BRCA1/2* gene status in women with healthy ovaries. From January 2013 to December 2017 a total of 255 patients addressed to germline *BRCA1/2* testing and pelvic US documenting normal ovaries, were retrospectively included. Feature selection for univariate analysis was carried out via correlation analysis. Multivariable analysis for classification of germline *BRCA1/2* status was then carried out via logistic regression, support vector machine, ensemble of decision trees and automated machine learning pipelines. Data were split into a training (75%) and a testing (25%) set. The four strategies obtained a similar performance in terms of accuracy on the testing set (from 0.54 of logistic regression to 0.64 of the auto-machine learning pipeline). Data coming from one of the tested US machine showed generally higher performances, particularly with the auto-machine learning pipeline (testing set specificity 0.87, negative predictive value 0.73, accuracy value 0.72 and 0.79 on training set). The study shows that a radiogenomics model on machine learning techniques is feasible and potentially useful for predicting g*BRCA1/2* status in women with healthy ovaries.

## Introduction

Breast cancer susceptibility genes (*BRCA1* and *BRCA2*) pathogenic variants (PVs) are correlated with a substantial elevated lifetime risk of developing breast (BC) and/or ovarian cancer (OC)^1–6^. In particular, germline BRCA1 pathogenic variant carriers (g-BRCA 1 PV) have a lifetime risk of 65–80% of developing BC and 37–62% of developing OC, while BRCA 2 pathogenic variant carriers (g-BRCA 2 PV) have a lifetime risk of 45–85% for BC and 11–23% for OC respectively^1–5^.

Healthy women resulting as g-BRCA 1/2 PV carriers are offered lifesaving prophylactic procedures and medications to prevent the onset of cancer^7,8^.

Many studies documented an OC reduction risk by 85% to 95% after risk-reducing salpingo-oophorectomy and a BC risk reduction of 90–95% after bilateral risk-reducing mastectomy^7–13^.

The prevalence in the general population of g*BRCA1/2* PV varies between 0.2 and 0.3%^14,15^, but the high penetrance of these PVs should be considered in relationship to patients familial history and age^16,17^. Around 10–20% of ovarian cancer and 6% breast cancer overall are caused by inheritable BRCA1/BRCA2 PVs^18^.

A population-based *BRCA* mutation screening has already been considered as too expensive^19^, therefore different strategies are needed for the future.

Current guidelines suggest that the assessment for cancer predisposition should be warranted only to those patients who are likely to have an inherited mutation, mainly based on personal or family history of BC or OR^11,17^.

Although this strategy is currently considered as the most cost-effective option, the available tools (such as BOADICEA, Myriad, BRCAPRO, etc.) combined with pre-test genetic counselling, showed a moderate to high diagnostic accuracy (around 2.6% diagnostic error rate) in predicting *gBRCA1/2* mutations, however, a number of individuals, potentially at risk, might not be identified^20–25^.

Demand for BRCA testing is increasing and in 2018, in the USA, the first direct-to-consumer test to report on *gBRCA1/2 PVs* was authorized^26^.

The technological development of analytical tools, able to generate and deal with large volumes of data, has been dramatically changing the overall scenario in the last years.

In particular, the possibility to perform quantitative analysis of medical imaging and its association with biological and clinical markers, such as gene expression, has led to a novel approach called “radiogenomics”^27–29^.

Pelvic ultrasound (US) scan is a widespread technique, regularly used in clinical practice to detect possible adnexal disorders. Normal fallopian tubes are not typically identified on US while exquisite images of the ovaries can be provided in both pre- and postmenopausal women^30^. BRCA-associated OCs seem to originate in the fallopian tube but there is some evidence that up to 21% of occult cancers may involve the ovary alone^31^.

If *gBRCA1/2 PV*s affect ovarian imaging features, it could be possible to develop a model to assess *gBRCA1/2* status through a radiogenomic analysis of US images of normal ovaries.

This model could help to improve patients’ selection for BRCA1/2 testing, overcoming the limits of the current criteria-based strategies.

We aimed at designing such a model and assessing its feasibility and performance.

## Methods

### Study design

This is an observational, single center study with patients retrospectively enrolled at the Fondazione Policlinico Universitario Agostino Gemelli IRCCS of Rome, Italy from January 2013 to December 2017.

A radiomics machine learning approach, based on features extracted from US images of the ovaries, was proposed to predict *gBRCA1/2* carrier status.

The retrospective data on *gBRCA1/2* testing performed on patients with NGS technique was considered as the reference standard of the radiomics analysis.

Transvaginal pelvic US was performed by dedicated gynecologic sonographers according to current international indications^32,33^.

This study was conducted in accordance with the declaration of Helsinki and was approved by the the Fondazione Policlinico Universitario Agostino Gemelli IRCCS ethics committee (Prot. 50543/19; ID: 2907), with the requirement for informed consent.

### Study population

The inclusion criteria were defined as follows: (i) patients recommended for BRCA testing according to international guidelines^7,17^ and (ii) patients who underwent *gBRCA1/2* testing in our institution (iii) and patients who underwent transvaginal pelvic US performed in our institution providing at least one picture of one healthy ovary and (iv) patient’s images had to be stored in .dicom format.

The exclusion criteria were (i) personal diagnosis of OC or (ii) ovarian abnormal findings (eg. ovarian endometriomas, dermoid cyst, ovarian cystoadenofibroma, ovarian borderline tumour…) but functional cysts at pelvic US or (iii) unavailable results of *gBRCA1/2* testing or (iv) refusal to provide written informed consent.

### *gBRCA1/2* testing data acquisition

Recommendations for genetic counseling were provided according to international guidelines^7,17^.

All patients included in the study received oncogenetic counseling before *BRCA* testing and signed written informed consent for *BRCA* analysis. Standardized procedures according to previously published workflows were observed^34,35^.

DNA for genotyping was isolated from whole blood samples by a non-automated method based on a commercial kit (Roche Diagnostics, Basel, Switzerland), and spectral analysis was performed using a NanoPhotometer (Implen, München, Germany). In order to achieve the highest efficiency in multiplex polymerase chain reaction (PCR), DNA was extracted just before the amplification step.

Finally, DNA samples showing concentrations of 15 ng/mL, low quality (A260/A280 of 1.7), or a ‘smeared profile’ were re-extracted.

Molecular analysis by massive parallel sequencing using the Illumina MiSeq system (Illumina Inc., San Diego, CA, USA) was then performed^36–40^.

In addition, horizon reference material was also used^36^ and the bioinformatics pipeline was validated using reference materials (EMQN scheme)^37^.

As previously reported, the bioinformatic “Amplicon Suite” CE- IVD tool (SmartSeq s.r.l., Novara, Italy) was used to determine depth of region covered, copy number status and variant calling^38^.

Variants with disrupting effects, such as frameshift, nonsense, canonical-splice site and such missense, along with large rearrangements, which were overall unequivocally reported as “pathogenic (class 5)”, within the main databases based on multiple lines of evidence and expert reviewing board (like ENIGMA Consortium) and the International Agency for research on Cancer (IARC) annotation, were assigned to the *gBRCA1/2* PV group.

All DNA samples resulting as negative for g*BRCA1/2* alterations at the massive parallel sequencing, were further analyzed by multiplex ligation dependent probe amplification (MLPA) or multiplex amplicon quantification assays^39^.

All amplicons with coverage under 38x, and all g*BRCA1/2* variants that were classified as damaging (class 5), probably damaging (class 4) and variants of uncertain significance (class 3, VUS) were confirmed by targeted Sanger sequencing. Finally, all positive results detected by MLPA on two independent DNA samples were confirmed by long-range PCR (Expand Long Template PCR System, Roche Applied Science)^40^.

### Pelvic US imaging protocol

All pelvic US examinations were performed by dedicated gynecologic oncologists with more than 10 years of experience in gynecological ultrasound. The examinations were carried out using 4 different high quality ultrasound equipment (GE Healthcare Voluson E10, Canon Toshiba Aplio-i900, Samsung Elite, Esaote-My LabTM Twice). Only transvaginal US probes were used to acquire images (7–10 MHz) using a standardized examination technique^41^.

When performing ovarian examination with real‐time 2D‐US, the patient was positioned in lithotomy position, with empty bladder and the operator scanned the ovary in both axial and coronal planes, to identify the best image.

The ovary had to occupy at least 50% of the screen along its largest axis.

Three orthogonal maximum diameters of the ovary (total length; antero-posterior measurement on the same scan; transverse length in a perpendicular image) for a maximum of 2 images per ovary were required, for a maximum of 2 images per ovary if possible. Current international indications were followed by all sonographers^32,33^. Other ultrasound features described were: morphology, echostructure and eventually abnormal vascularitation. The presence of functional cysts or the absence of one ovary, were was not considered an exclusion criterion. The presence of ovarian tumors, benign, uncertain or suspicious for malignant, according to the IOTA classification led to the exclusion from the current analysis^41^. In case of artifact, such as bowel gas shadowing, the patients were excluded if the images were not clear for analysis.

### Image postprocessing

Code for the project is available at https://github.com/kbolab/PROBE. The repository contains the scripts for all the different part of the analysis, from ROI extraction, to feature computation, to statistical analysis. Everything regarding radiomics processing is a direct porting of the R library Moddicom.

**Figure 1:**
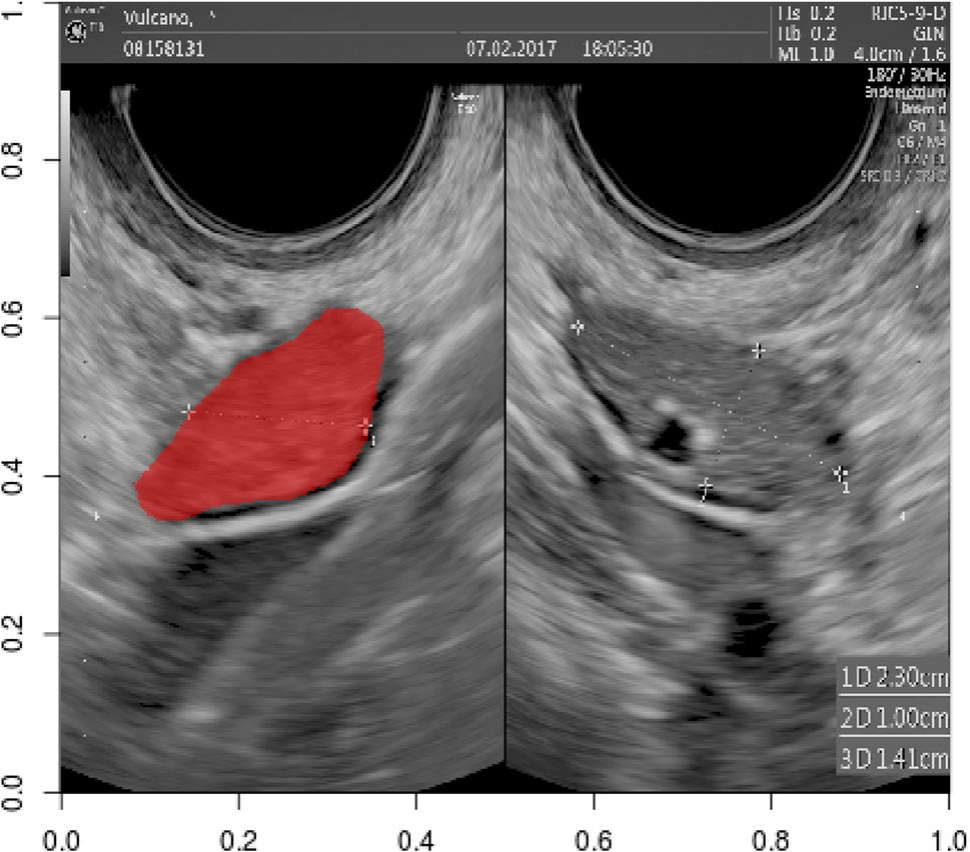
A ROI mask applied to the image used to extract the pixel values (three orthogonal diameters of left ovary).

The regions of interest (ROI) were manually delineated on the US image via the software Aliza version 1.48 by a trained gynecologist (Fig. 1)^42^.

Guidelines describing how to define the boundaries of the ovary were provided before starting ROI delineation and two senior dedicated gynecologic sonographers with at least 10 years’ experience in pelvic US imaging were responsible for the evaluation and independent check of ovarian segmentation.

Since the BRCA status outcome was associated to the patient, and each patient could be associated with a number of images varying from 1 to 4, the corresponding DICOM folder structure was organized in a hierarchical way so that a single ROI was labeled with the following set of tags: US machine, patient ID, left or right ovary, longitudinal or coronal plane. Each leaf folder structure contained therefore two files: the original DICOM and the ROI mask DICOM.

All the image files had been previously anonymized according to the existing privacy regulations.

### US radiomics feature extraction and validation

The original DICOM file and the corresponding ROI segmentation masks were uploaded on the MODDICOM platform, an in-house developed software for radiomics analysis^43^.

The software extracted the pixel values contained inside the ROI boundaries and computed 232 radiomics features. Prior to feature extraction, all the ROIs were rescaled to pixel value histograms between 0 and 255.

After that, four groups of imaging features were extracted from each normalized US image with manually segmented ROIs: 20 first order statistics, 14 morphological, 183 texture and 15 fractal features. First order statistics features describe the properties of the image grey level histogram considered as a whole. Morphological features describe the properties of the ROI in space. Texture features describe the properties of the local distribution of grey levels inside the ROI. Fractal features compute the fractal dimension through the box-counting algorithm. The software implementation, was entirely validated within the Image Biomarker Standardization Initiative^44^ to ensure reproducibility and methodological robustness of the radiomics features extraction pipeline.

### Statistical analysis

#### Feature-outcome association in univariate analysis

Radiomics features are often strongly correlated with each other, so that one can identify clusters of similarly distributed features through the pair-wise Pearson correlation test.

This approach is useful to reduce features dimensionality; the higher the number of features tested against an outcome, the higher the probability of over-fitting the data or getting a significant result just by chance will be^45^.

The feature selection for univariate analysis was carried out via correlation analysis to remove all but one feature among groups of highly correlated features by setting a Pearson correlation threshold of 0.9. The remaining features were tested for association with the outcome with the Wilcoxon–Mann–Whitney test. A p-value of 0.05 was considered as statistically significant.

The number of significant test results was then compared to the expected number of type I errors to account for multiple testing^46^.

#### Machine learning-based models

Multivariable analysis for classification of *gBRCA1/2* status was carried out via four strategies involving machine learning techniques: logistic regression (A), support vector machine (B), ensemble of decision trees (C) and automated machine learning pipelines (D). The corresponding models were trained on the 75% of the data, while the remaining 25% of data was left out for testing purposes.

A first feature selection was carried out for each strategy with correlation analysis to remove all but one highly correlated feature by setting a Pearson correlation threshold of 0.9, as performed in the univariate analysis.

Strategies A and B involved logistic regression and radial kernel support vector machine, respectively: both strategies had a forward feature selection based Cohen Kappa values at the 0.75 splitted training set ROC Youden index.

We used Cohen’s Kappa as confusion matrix evaluation metrics to take into account the class imbalance and the related fact that expected accuracy is “skewed” towards values higher than 50%. Also, for the same reason, this metric allows for direct comparison between results obtained on datasets with different class distribution, so that for future work on external validation or prospective data the classification performances will be directly comparable with the ones reported in this paper.

Detailed steps of these iterative strategies, starting from *n* equals to 1, are:

i. All the possible different models with *n* features are trained using all the different groups of *n* features;
ii. The ROC curve on the training set is computed for every *n* feature model;
iii. The optimal cut-off point according to Youden index will be found on every ROC curve of ii) and the Cohen Kappa value of the classification matrix at the optimal point will be computed;
iv. The model with the highest value of the Cohen Kappa computed in iii) is then held for the next iteration, while all the others are discarded;
v. All the possible different models with *n* + 1 features are trained adding to the *n* features selected in the previous step, all the features left available, one by one;
vi. The process is then repeated until the Cohen’s Kappa value does not increase by adding a new feature to the model.

Strategy C involved Extreme Gradient Boosting (XGBoost)^46^ with extensive grid search on the hyperparameters space and fivefold cross validation error on the 0.75 split training set as the metric to be minimized. The selected model was then tested on the remaining 25% of data.

Auto Machine Learning for classification with TPOT Python library^47^ has been used in strategy D, testing the spectrum of classification models and their hyperparameters with a heuristic search conducted through a genetic algorithm with the fivefold cross validation error on the 0.75 splitted training set as the metric to be minimized.

The genetic algorithm was initialized with TPOT default parameters. The selected model was then tested on the remaining 25% of data.

All statistical analyses were conducted with R version 3.4.4^48^ and Python version 3.7.4^49^.

## Results

### Clinical characteristics of the study cohort

From January 2013 to December 2017, 1865 consecutive patients underwent *gBRCA 1/2* testing in our institution. Among these women, 248 had a *gBRCA1* PV or VUS (13.3%), 231 had a *gBRCA2* PV or VUS (12.3%), 3 had *gBRCA-1 and -2* PVs (compound heterozygotes, 0.16%) while 1383 were wild type (WT) (74%).

Patients with personal history of OC and/or endometrial cancer (109) were excluded.

Moreover, 684 patients out of 1756 underwent pelvic US at our institution. Pelvic US was part of the annual gynecologic assessment worldwide recommended for the optimal promotion of women’s health^50^.

Patients who did not have US images stored as *.dicom* files and those with abnormal findings regarding the ovaries at US imaging were excluded from the study. Even in cases of unilateral lesion, we preferred not to use for analysis the contra lateral ovary in order to reduce possible bias.

A total of 890 images was finally analyzed in the current study.

**Figure 2:**
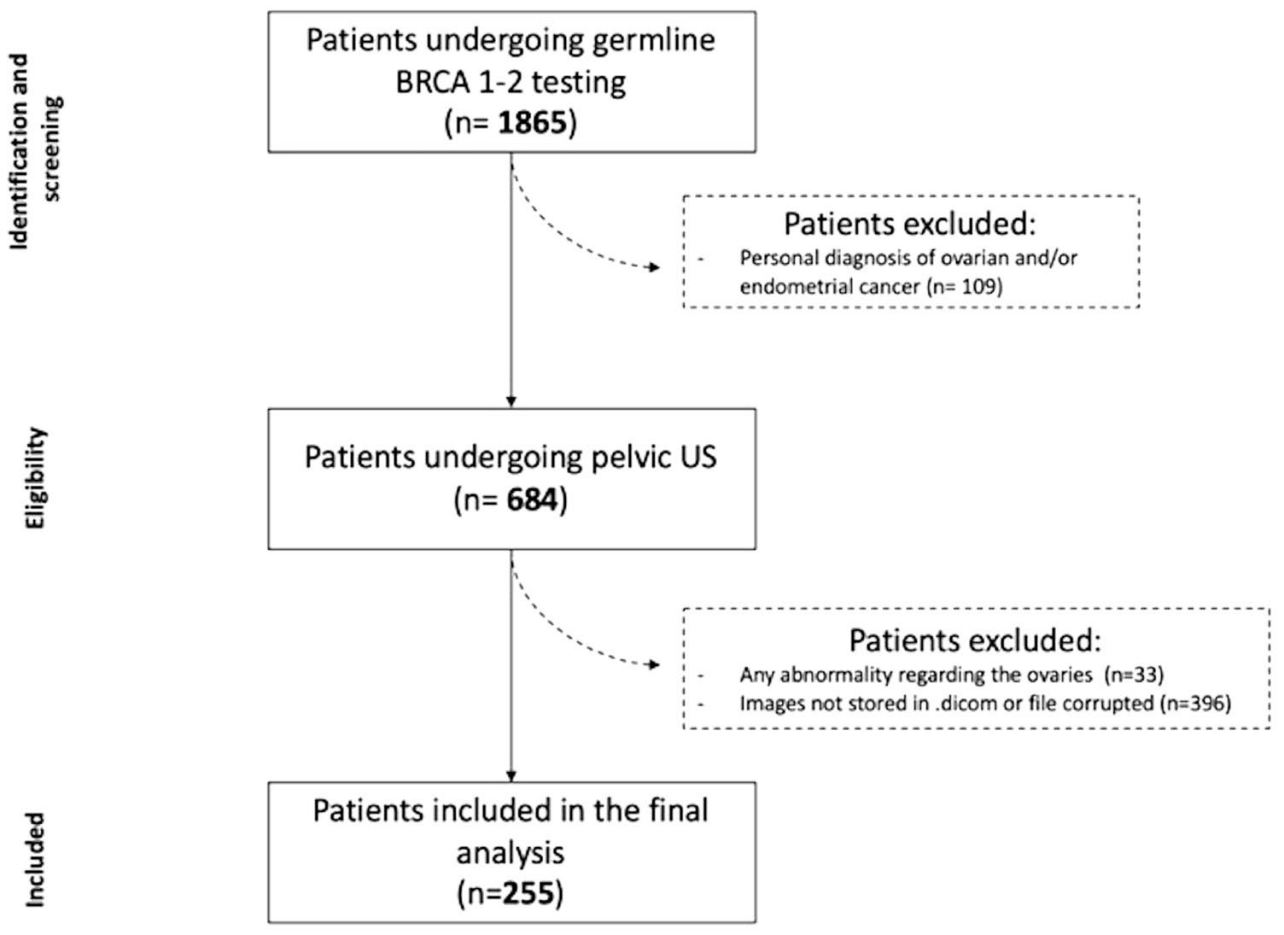
Flow chart of the study.

Figure 2 illustrates the general flowchart of the study.

Table 1 reports the clinical characteristics of the 255 included women; no significant differences were found in most clinico-pathologic factors between the testing and training sets.

**Table 1:** The clinical characteristics of the testing and training dataset.

	Overall (n = 255)	Testing dataset (n = 64)		Training dataset (n = 191)		*p* value
		g-BRCA 1–2 p	g-BRCA WT	g-BRCA 1–2 p	g-BRCA WT	
Number (%)	255 (100)	25 (39)	39 (61)	73 (33)	118 (67)	1.000
Mean age (year)	45.5	45	46	47	44	0.878
Age range (year)	23–79	23–73		26–79		
**Hormonal status**						
Fertile, no E/P	92 (36)	8 (32)	7 (18)	30 (41.1)	47 (39.8)	0.454
Iatrogenic menopause	97 (38)	9 (36)	23 (59)	18 (24.6)	47 (39.8)	1.000
Menopause	61 (24)	7 (28)	7 (18)	24 (32.9)	22 (18.7)	0.941
HT in menopause	1 (0.4)	–	1 (2.5)	–	–	–
Unknown	4 (1,6)	1 (4)	1 (2.5)	1 (1.4)	2 (1.7)	–
**Reason to access** ***BRCA*** **testing**						
Family history of cancer	45 (17.5)	6 (24)	2 (5.1)	24 (32.9)	12 (10.2)	0.460
Personal history of cancer	204 (80)	19 (76)	35 (89.7)	49 (67.1)	102 (86.4)	0.843
Unknown	6 (2.5)	–	2 (5.2)	–	4 (3.4)	–
**Previous ovarian surgery (benign findings)**						
Yes	15 (6)	1 (4)	3 (7.7)	7 (9.6)	4 (3.4)	0.458
No	240 (94)	24 (96)	36 (92.3)	66 (90.4)	114 (96.6)	0.758
**US machine**						
GE Healthcare Voluson E10	101 (40)	7 (28)	19 (48.7)	29 (39.7)	47 (40)	0.425
Canon Toshiba Aplio-i900	53 (21)	4 (16)	9 (23.1)	17 (23.3)	23 (20)	0.670
Samsung Elite	62 (24)	10 (40)	5 (12.8)	20 (27.4)	27 (23)	0.183
Esaote-My LabTM Twice	37 (15)	4 (16)	6 (15.4)	7 (9.6)	20 (17)	0.669
**Number of images/patient**						
1	5 (1.9)	0 (0.0)	1 (2.5)	4 (5.4)	0 (0.0)	0.401
2	42 (16.5)	4 (16.0)	6 (15.3)	14 (19.2)	18 (15.2)	1.000
3	36 (14.1)	3 (12.0)	6 (15.3)	6 (8.2)	21 (17.8)	0.824
4	172 (67.4)	18 (72.0)	26 (66.7)	49 (67.1)	79 (66.9)	0.897
**Ovarian meas. max (mean, mm)**						
Right	22.1	21.3	18.3	21.9	23.6	0.771
Left	20.4	20.7	18.7	19.2	21.6	0.794
**Ovarian volume (mean, mm**^**3**^**)**						
Right	4878.0	3545.7	2973.3	7138.3	4536.2	0.340
Left	4228.8	3787.6	3045.4	3715.9	4998.9	0.788

Overall, the mean age was 45.5 years but the majority of women (62%) were in menopause (both iatrogenic or natural). This is consistent with the fact that the indication to the test was mainly related to personal history of BC (80%). The mean number of images per patient was 3.5, ensuring quality to US approach.

No significant differences were observed in the two image sets**.**

### Correlation and univariate analysis

After the segmentation of the ROIs and the following feature extraction, the final feature set was composed by 232 radiomics features.

The outcome was binarized into *gBRCA1/2* WT patients (558 images) and *gBRCA1/2-ve* patients (332 images). *gBRCA 1/2 VUS* were considered among *gBRCA 1/2* PVs because although the pathogenetic potential of this group of mutations is unknown, these patients deserve to be tested and followed up.

A specific dataset for each US machine was also produced for the analysis with the following number of images: Voluson (350 images), Toshiba (190 images), Samsung (221 images), Esaote (134 images).

The proportion of *g-BRCA 1-2* pathogenetic cases was comparable in all the datasets.

A first feature selection was carried out via correlation analysis on each dataset to reduce the feature space at a Pearson correlation threshold of 0.9, resulting in 61 features for the full dataset, 45 for Voluson, 42 for Toshiba, 44 for Samsung and 40 for Esaote.

The overlapping features identified from each machine after Pearson correlation analysis are shown in Supplementary Table 1.

The radiomics features in these sorted datasets were tested individually in univariate analysis using Wilcoxon–Mann–Whitney test with the binary outcome.

The features which resulted in a statistically significant test were 6 for the full dataset, 16 for the Voluson dataset, 3 for the Toshiba dataset, 3 for the Samsung dataset and 2 for the Esaote dataset. As a percentage of the tested features, these figures represent the 9.8%, 35.0%, 7.1%, 6.8% and 5.0% respectively (as shown in Table 2).

**Table 2:** Number of features selected excluding (also, in percentage of the number of selected features).

Dataset	Number of features selected with Pearson correlation test	Number of features selected with Wilcoxon–Mann–Whitney test
All	61	6 (9.8%)
Voluson	45	16 (35%)
Toshiba	42	3 (7.1%)
Samsung	44	3 (6.8%)
Esaote	40	2 (5.0%)

The overlapping statistically significant features identified from each machine after Wilcoxon–Mann–Whitney test, are shown in Supplementary Table 2.

Since the significance threshold was set at 0.05, we observed that the number of significant features was above the expected value of type I errors in four out five datasets, with a particularly high rate of statistically significant associations for the features coming from Voluson images.

The p-values of the Wilcoxon–Mann–Whitney test for the significant features are reported in Table 3.

**Table 3:** Radiomics features whose association test with the binary outcome was statistically significative with corresponding p-value, for the different datasets.

US type	Feature name	*gBRCA* mutation univariate test p-value
All	F_cm.info.corr.2	2.00 e−04
All	F_cm.joint.var	2.55 e−02
All	L_major	2.72 e−02
All	F_rlm.gl.var	3.02 e−02
All	F_cm.sum.entr	3.03 e−02
All	FD_0.100	3.40 e−02
Voluson	F_cm.info.corr.2	3.24 e−06
Voluson	F_cm.inv.diff	7.93 e−06
Voluson	F_cm.sum.avg	9.36 e−06
Voluson	F_cm.inv.diff.norm	4.13 e−05
Voluson	F_rlm.sre	4.42 e−04
Voluson	F_cm.energy	5.38 e−04
Voluson	F_rlm.lrlrlm	6.70 e−04
Voluson	F_rlm.lgre	1.05 e−03
Voluson	F_rlm.srlge	1.62 e−03
Voluson	F_cm.joint.max	6.80 e−03
Voluson	F_rlm.lrhge	6.85 e−03
Voluson	F_szm.sze	7.42 e−03
Voluson	F_szm.zsnu.norm	9.19 e−03
Voluson	F_cm.corr	1.36 e−02
Voluson	F_cm.clust.prom	2.38 e−02
Voluson	FD_0.100	2.60 e−02
Toshiba	F_stat.range	2.81 e−03
Toshiba	F_rlm.rl.entr	3.79 e−02
Toshiba	F_szm.lze	4.38 e−02
Samsung	F_morph.surface	7.20 e−03
Samsung	F_szm.z.entr	1.46 e−02
Samsung	F_rlm.glnu	4.54 e−02
Esaote	F_cm.2.5Dmerged.corr	1.80 e−02
Esaote	F_cm.clust.prom	4.58 e−02

The vast majority of statistically significant features were texture features: 15 out of 16 for Voluson; 2 out of 2 for Esaote; 2 out of 3 for Samsung; 2 out of 3 for Toshiba. Among these, the most selected texture type of features were co-occurrence matrix and run-length matrix based features.

### Performance of machine learning models

**Figure 3:**
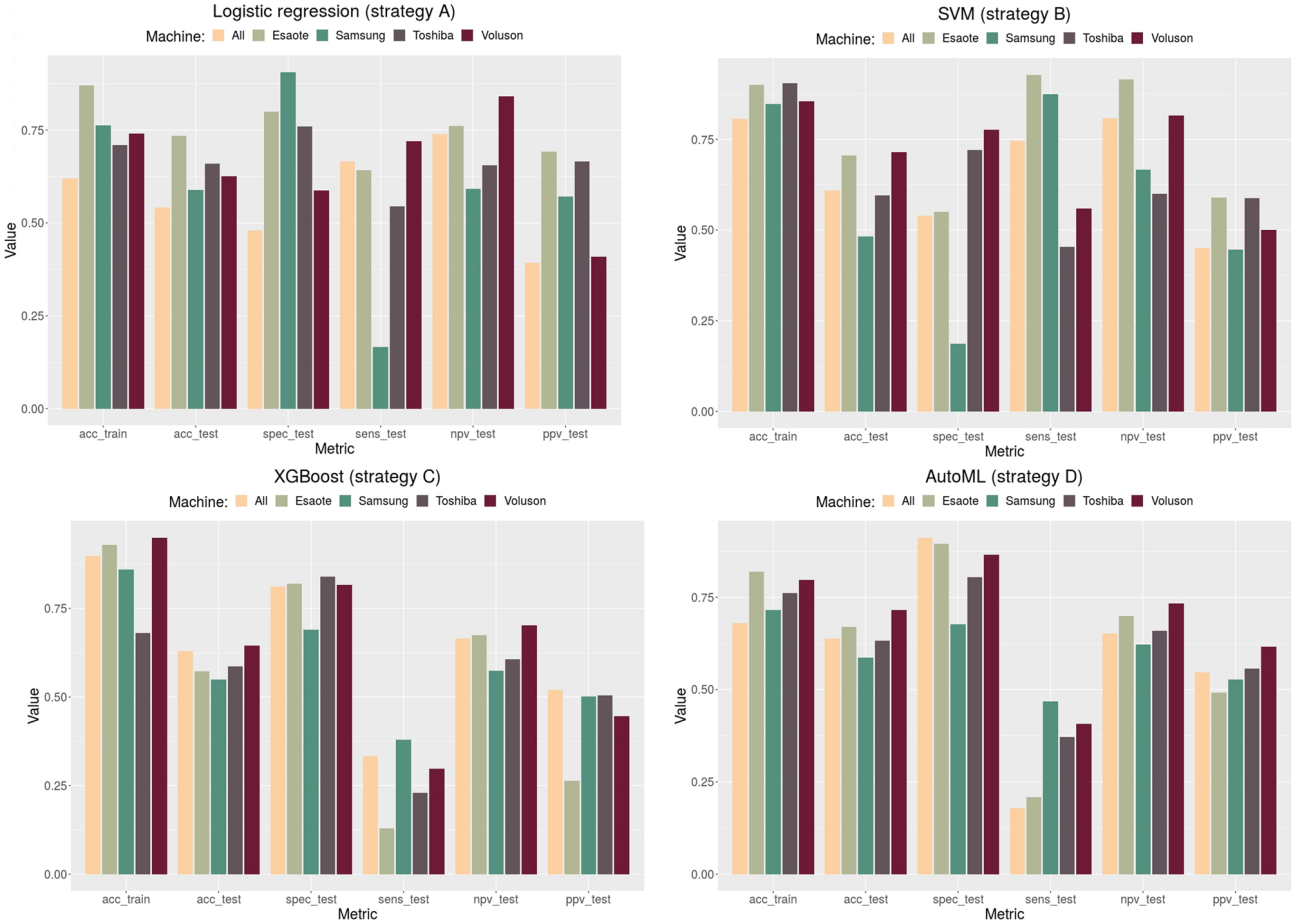
Different machine learning models performance for the different datasets. Metrics from left to right : accuracy on training set, accuracy on testing set, specificity on testing set, sensitivity on testing set, negative predictive value on testing set, positive predictive value on testing set.

Multivariable analysis for *gBRCA* pathogenetic variant classification was carried out with the four described machine learning strategies (A), (B), (C), and (D). Classification metrics for the different models on the five datasets are reported in Supplementary Table 3 and summarized in Fig. 3. The train-test split was per patient so to prevent information leakage from training to testing set. After the split, the model was trained and tested per image, and its diagnostic performance is reported on an image basis.

Considering the full dataset, the four strategies obtained a similar performance in terms of classification accuracy on the testing set, varying from 0.54 of logistic regression of strategy A to 0.64 of strategy D with the auto-machine learning pipeline.

Strategy D showed also the highest value of specificity on the testing set (0.91) and a negative predictive value of 0.65, comparable to the one obtained with strategy C but lower than the corresponding values obtained with strategies A and B, which were 0.74 and 0.80, respectively.

Considering the Voluson data only, performances were generally higher: in particular, testing set specificity and negative predictive value reached 0.77 and 0.81, respectively, with the radial kernel support vector machine of strategy B; 0.82 and 0.70 with the extreme gradient boosting of strategy C, and 0.87 and 0.73 with the auto-machine learning pipeline of strategy D.

The latter combination, strategy D on Voluson data, showed also the best consistency between training and testing set accuracy (0.79 and 0.72 respectively) suggesting that the adopted strategy performance was particularly robust to avoid data overfitting, which might improve the classification performances on larger image numbers to obtain both a higher specificity and a higher negative predictive value.

## Discussion

In this single center study, we developed an automated machine learning pipeline model with encouraging performances to identify *gBRCA1/2* status based on US images of healthy ovaries, acquired on different US machines.

This result can be considered as an instance of “real world data” from the perspective of radiomics analysis and paves the way to possible developments of this model with direct consequences on patients diagnostic workflow and prognostic stratification, in the frame of the most modern personalized medicine paradigms.

More specifically, the described model, as a large-scale screening test, would assign only 13 out of 100 *gBRCA1/2* WT patients (with the current value of specificity at 0.87) to unnecessary genetic screening.

In our series, 157 out of 255 patients (61.5%) were assigned to genetic screening based on current clinical selection criteria, but resulted as *gBRCA1/2 WT*.

Not many data have been published on the performance of the current clinical criteria selection in detecting BRCA1/BRCA2 mutations. Nevertheless, as reported by Manchanda et al.^18^, despite 25 years of BRCA testing and effective mechanisms for prevention, current guidelines and access to testing/treatment pathways remain complex and associated with a massive under-utilisation of genetic testing. Only 20% of eligible US women have accessed/undergone genetic testing. A UK study showed the large majority (> 97%) of BRCA carriers in the population remain unidentified^51^. In addition, current approaches use established clinical-criteria/family-history based a priori BRCA probability thresholds to identify high-risk individuals eligible for BRCA testing. The estimation of likelihood of being carrier of BRCA1/2 PV is based on different tools, with possible selection biases related to different algorithms performing the risk assessment: in such countries the threshold is 20% while in others 10%. Over 50% BRCA carriers do not fulfil clinical criteria and are therefore missed^52^.

Our model, when tested and validated on the general population, could be an extremely cost-effective strategy for a population-based BRCA mutation screening.

Nevertheless, the current described negative predictive value of 0.73 implies that 27 out of 100 *gBRCA1/2* PV carriers would not be assigned to the needed genetic test, probably representing the most significant limit of this hypothesis generating radiogenomics approach.

Compared to CT or MRI, radiomics on US images is a relatively new and less explored type of analysis. Nonetheless, it is gaining gound^53–56^.

Most significant features for the screening tool both in univariate analysis and in the machine learning models are texture features, which are often not quite easy to visualize. Nevertheless, among the most influential features (especially considering the Voluson dataset) those related to clusters of high-intensity grey levels are predominant. In particular, the “short run high grey level emphasis” feature is the leading feature in the xgboost model with roughly twice the “gain” value than the second most important feature. Also, the univariate statistical association of this feature with the binary outcome through Wilcoxon–Mann–Whitney test is significative with a p-value of order 10^−5^, the sign of the effect being that BRCA mutated group have an higher feature median value (i.e. BRCA mutated images have more short runs, or small clusters, of high grey level intensities).

As previously discussed, Voluson machine showed more significant features and higher performances compared with the other machines. The explanation may lie in the fact that Voluson dataset has 60% more records than the biggest among Samsung, Esaote, and Toshiba datasets (which is the Samsung dataset with 260 records). This can cause stronger statistical associations if the effect is there. However, more data with a balanced number of images among various US machines are needed to clarify this aspect.

Based on this analysis, authors believe that this model deserves further investigation to increase its performance and to better describe its translational application in daily clinical practice.

Several issues and limitations have been encountered during the analysis.

First, the retrospective nature of the study represents an unavoidable source of selection bias and imaging data inhomogeneity. In particular, patients were not divided in subgroups according to their hormonal status and for fertile age women (36%) the time on which the ultrasound was performed was not established. All these aspects may have impacted on textural radiomic features.

Second, the monocentric nature of the study and the qualification of our institution as a referral center for US in gynecological malignancies may not fully reflect real word data, especially in terms of US image quality.

Third, the absence of an external validation does not allow us to draw any definitive conclusion on the replicability of the model, even if the impact of this limit is reduced thanks to the heterogeneity of the tested population and the use of different US scanners.

Fourth, our patients were only screened for *BRCA1/2*: no other genes involved in the same homologous recombination repair pathways were included in the analysis. Therefore, we cannot exclude the presence of PVs in the other genes whose mutational status could correlate with US patterns, limiting the clinical impact of the proposed approach.

Fifth, the model was developed on women with high risk factors for *gBRCA1/2* PVs therefore could be applied only to this clinical setting.

These aspects will be taken into account at best to improve the power of our model in following investigations.

Overall, the model could help patients who may have unnecessary genetic tests when identified by the clinical screening method. However, its actual performance, in terms of negative predictive values, does not allow applying it as a reliable screening tool to optimize patient selection for genetic screening of *BRCA1/2* genes.

However, the novelty of this radiogenomics application, its potentiality as a screening tool and the encouraging preliminary results support the reliability and the value of our findings.

For these reasons, we are planning to expand the series including patients enrolled in 2018 and 2019, validate the model on external cohorts of patients and finally test it in the general population as a screening tool.

In conclusion, the authors believe that in the next future a radiogenomics approach based on ovarian US images can become a successful screening tool to identify women who are highly likely to have *gBRCA1/2* PV and therefore should be assigned to genetic testing and counselling pursuing healthcare cost-containment policies.

This radiogenomic model could represent a cost-effective, highly reproducible**,** large-scale extendable and time saving screening tool although further investigations are necessary to improve sensitivity and specificity before its effective clinical release.

## Supplementary information

Supplementary Information.

## References

[1] Antoniou A, Pharoah PD, Narod S (2003). Average risks of breast and ovarian cancer associated with BRCA1 or BRCA2 mutations detected in case series unselected for family history: A combined analysis of 22 studies. Am. J. Hum. Genet..

[2] Chen S, Parmigiani G (2007). Meta-analysis of BRCA1 and BRCA2 penetrance. J. Clin. Oncol..

[3] Mavaddat N, Peock S, Frost D (2013). Cancer risks for BRCA1 and BRCA2 mutation carriers: Results from prospective analysis of EMBRACE. J. Natl. Cancer Inst..

[4] Hartmann LC, Lindor NM (2016). Risk-reducing surgery in hereditary breast and ovarian cancer. N. Engl. J. Med..

[5] Balmaña J, Díez O, Castiglione M, ESMO Guidelines Working Group (2009). BRCA in breast cancer: ESMO clinical recommendations. Ann. Oncol..

[6] Torre LA, Bray F, Siegel RL, Ferlay J, Lortet-Tieulent J, Jemal A (2015). Global cancer statistics, 2012. CA Cancer J Clin..

[7] National Comprehensive Cancer Network. Genetic/Familial High-Risk Assessment: Breast and Ovarian. NCCN Clinical Practice Guidelines in Oncology. Version 3.2019—January 18, 2019

[8] Walker JL, Powell CB, Chen LM, Carter J, Bae Jump VL, Parker LP, Borowsky ME, Gibb RK (2015). Society of Gynecologic Oncology recommendations for the prevention of ovarian cancer. Cancer.

[9] Rebbeck TR, Kauff ND, Domchek SM (2009). Meta-analysis of risk reduction estimates associated with risk-reducing salpingo-oophorectomy in BRCA1 or BRCA2 mutation carriers. J. Natl. Cancer Inst..

[10] Domchek SM, Friebel TM, Neuhausen SL, Wagner T, Evans G, Isaacs C, Garber JE, Daly MB, Eeles R, Matloff E, Tomlinson GE, Van’t Veer L, Lynch HT, Olopade OI, Weber BL, Rebbeck TR (2006). Mortality after bilateral salpingo-oophorectomy in BRCA1 and BRCA2 mutation carriers: A prospective cohort study. Lancet Oncol..

[11] Eleje GU, Eke AC, Ezebialu IU, Ikechebelu JI, Ugwu EO, Okonkwo OO (2018). Risk-reducing bilateral salpingo-oophorectomy in women with BRCA1 or BRCA2 mutations. Cochrane Database Syst. Rev..

[12] Ludwig KK, Neuner J, Butler A, Geurts JL, Kong AL (2016). Risk reduction and survival benefit of prophylactic surgery in BRCA mutation carriers, a systematic review. Am. J. Surg..

[13] Mann GJ, Thorne H, Balleine RL, Butow PN, Clarke CL, Edkins E, Evans GM, Fereday S, Haan E, Gattas M, Giles GG, Goldblatt J, Hopper JL, Kirk J, Leary JA, Lindeman G, Niedermayr E, Phillips KA, Picken S, Pupo GM, Saunders C, Scott CL, Spurdle AB, Suthers G, Tucker K, Chenevix-Trench G, Kathleen Cuningham Consortium for Research in Familial Breast Cancer (2006). Analysis of cancer risk and BRCA1 and BRCA2 mutation prevalence in the kConFab familial breast cancer resource. Breast Cancer Res..

[14] Ferla R, Calò V, Cascio S, Rinaldi G, Badalamenti G, Carreca I, Surmacz E, Colucci G, Bazan V, Russo A (2007). Founder mutations in BRCA1 and BRCA2 genes. Ann. Oncol..

[15] Nelson, H.D., Fu, R., Goddard, K. *et al.*Risk Assessment, Genetic Counseling, and Genetic Testing for BRCA-Related Cancer: Systematic Review to Update the U.S. Preventive Services Task Force Recommendation. Report No.: 12-05164-EF-1. (Agency for Healthcare Research and Quality (US), Rockville, 2013). 24432435

[16] Kuchenbaecker KB, Hopper JL, Barnes DR, Phillips KA, Mooij TM, Roos-Blom MJ, Jervis S, van Leeuwen FE, Milne RL, Andrieu N, Goldgar DE, Terry MB, Rookus MA, Easton DF, Antoniou AC, McGuffog L, Evans DG, Barrowdale D, Frost D, Adlard J, Ong KR, Izatt L, Tischkowitz M, Eeles R, Davidson R, Hodgson S, Ellis S, Nogues C, Lasset C, Stoppa-Lyonnet D, Fricker JP, Faivre L, Berthet P, Hooning MJ, van der Kolk LE, Kets CM, Adank MA, John EM, Chung WK, Andrulis IL, Southey M, Daly MB, Buys SS, Osorio A, Engel C, Kast K, Schmutzler RK, Caldes T, Jakubowska A, Simard J, Friedlander ML, McLachlan SA, Machackova E, Foretova L, Tan YY, Singer CF, Olah E, Gerdes AM, Arver B, Olsson H, BRCA1 and BRCA2 Cohort Consortium (2017). Risks of breast, ovarian, and contralateral breast cancer for BRCA1 and BRCA2 mutation carriers. JAMA.

[17] Paluch-Shimon S, Cardoso F, Sessa C (2016). Prevention and screening in BRCA mutation carriers and other breast/ovarian hereditary cancer syndromes: ESMO Clinical Practice Guidelines for cancer prevention and screening. Ann. Oncol..

[18] Gaba F, Blyuss O, Liu X, Goyal S, Lahoti N, Chandrasekaran D, Kurzer M, Kalsi J, Sanderson S, Lanceley A, Ahmed M (2020). Population study of ovarian cancer risk prediction for targeted screening and prevention. Cancers..

[19] D’Andrea E, Marzuillo C, De Vito C (2016). Which BRCA genetic testing programs are ready for implementation in health care? A systematic review of economic evaluations. Genet. Med..

[20] Nelson HD, Pappas M, Cantor A, Haney E, Holmes R (2019). Risk assessment, genetic counseling, and genetic testing for BRCA-related cancer in women: Updated evidence report and systematic review for the US Preventive Services Task Force. JAMA.

[21] Tuffaha HW, Mitchell A, Ward RL, Connelly L, Butler JRG, Norris S, Scuffham PA (2018). Cost-effectiveness analysis of germ-line BRCA testing in women with breast cancer and cascade testing in family members of mutation carriers. Genet. Med..

[22] Anglian Breast Cancer Study Group (2000). Prevalence and penetrance of BRCA1 and BRCA2 mutations in a population-based series of breast cancer cases. Br. J. Cancer..

[23] Wood ME, Flynn BS, Stockdale A (2013). Primary care physician management, referral, and relations with specialists concerning patients at risk for cancer due to family history. Public Health Genomics..

[24] Manickam K, Buchanan AH, Schwartz ML, Hallquist ML, Williams JL, Rahm AK, Rocha H, Savatt JM, Evans AE, Butry LM, Lazzeri AL (2018). Exome sequencing-based screening for BRCA1/2 expected pathogenic variants among adult biobank participants. JAMA Netw. Open..

[25] Ellison G, Wallace A, Kohlmann A, Patton S (2017). A comparative study of germline BRCA1 and BRCA2 mutation screening methods in use in 20 European clinical diagnostic laboratories. Br. J. Cancer.

[26] U.S Food and Drug Administration. FDA authorizes, with special controls, direct-to-consumer test that reports three mutations in the BRCA breast cancer genes [news release]. Silver Spring (MD): FDA; (2018). https://www.fda.gov/NewsEvents/Newsroom/PressAnnouncements/ucm599560.htm. Accessed 7 May 2018.

[27] Story MD, Durante M (2018). Radiogenomics. Med. Phys..

[28] Bodalal Z, Trebeschi S, Beets-Tan R (2018). Radiomics: A critical step towards integrated healthcare. Insights Imaging..

[29] Aerts HJ, Velazquez ER, Leijenaar RT, Parmar C, Grossmann P, Carvalho S, Bussink J, Monshouwer R, Haibe-Kains B, Rietveld D, Hoebers F, Rietbergen MM, Leemans CR, Dekker A, Quackenbush J, Gillies RJ, Lambin P (2014). Decoding tumour phenotype by noninvasive imaging using a quantitative radiomics approach [Erratum in: Nat Commun. 2014;5:4644. Cavalho, Sara (corrected to Carvalho, Sara)]. Nat. Commun..

[30] Panchal S, Nagori C (2014). Imaging techniques for assessment of tubal status. J. Hum. Reprod. Sci..

[31] Yates MS, Meyer LA, Deavers MT, Daniels MS, Keeler ER, Mok SC (2011). Microscopic and early-stage ovarian cancers in BRCA1/2 mutation carriers: Building a model for early BRCA-associated tumorigenesis. Cancer Prev. Res..

[32] Coelho Neto MA, Ludwin A, Borrell A, Benacerraf B, Dewailly D, da Silva CF, Condous G, Alcazar JL, Jokubkiene L, Guerriero S, Van den Bosch T, Martins WP (2018). Counting ovarian antral follicles by ultrasound: A practical guide. Ultrasound Obstet Gynecol..

[33] Abuhamad A, Minton KK, Benson CB, Chudleigh T, Crites L, Doubilet PM, Driggers R, Lee W, Mann KV, Perez JJ, Rose NC, Simpson LL, Tabor A, Benacerraf BR (2018). Obstetric and gynecologic ultrasound curriculum and competency assessment in residency training programs: Consensus report. J. Ultrasound Med..

[34] Capoluongo E, Ellison G, Lopez-Guerrero JA (2017). Guidance statement on BRCA1/2 tumor testing in ovarian cancer patients. Semin. Oncol..

[35] Capoluongo E (2016). BRCA to the future: Towards best testing practice in the era of personalised healthcare. Eur. J. Hum. Genet..

[36] BRCA Germline I Reference Standard gDNA. https://www.horizondiscovery.com/brca-germline-i-hd793

[37] https://www.emqn.org/schemes/breast-ovarian-cancer-familial-full-version/

[38] Concolino P, Capoluongo E (2019). Genetic test reports were collected as part of the medical record. For the purpose of the study the outcome was binarized: g- BRCA1-2 p and VUS vs g-BRCA 1-2 wild type (g-BRCA WT). Expert Rev. Mol. Diagn..

[39] Minucci A, Scambia G, Santonocito C (2015). Clinical impact on ovarian cancer patients of massive parallel sequencing for BRCA mutation detection: The experience at Gemelli hospital and a literature review. Expert Rev. Mol. Diagn..

[40] Concolino P, Costella A, Minucci A, International Ovarian Tumor Analysis (IOTA) Group (2014). A preliminary quality control (QC) for next-generation sequencing (NGS) library evaluation turns out to be a very useful tool for a rapid detection of BRCA1/2 deleterious mutations. Clin. Chim. Acta..

[41] Timmerman D, Valentin L, Bourne TH, Collins WP, Verrelst H, Vergote I (2000). Terms, definitions and measurements to describe the sonographic features of adnexal tumors: A consensus opinion from the International Ovarian Tumor Analysis (IOTA) Group. Ultrasound Obstet. Gynecol..

[42] https://www.aliza-dicom-viewer.com/

[43] Dinapoli, N. *et al.* Moddicom: a complete and easily accessible library for prognostic evaluations relying on image features. In *Conference Proceeding IEEE Engineering in Medicine and Biology Society* 771–774. (2015) 10.1109/EMBC.2015.7318476 26736376

[44] Zwanenburg A, Vallières M, Abdalah MA (2020). The image biomarker standardization initiative: Standardized quantitative radiomics for high-throughput image-based phenotyping. Radiology.

[45] Park JE, Park SY, Kim HJ, Kim HS (2019). Reproducibility and generalizability in radiomics modeling: Possible strategies in radiologic and statistical perspectives. Korean J. Radiol..

[46] Chen, T. & Guestrin, C. XGBoost: A scalable tree boosting system. In *22nd SIGKDD Conference on Knowledge Discovery and Data Mining* (2016)

[47] Olson, R.S., Urbanowicz, R.J., Andrews, P.C., Lavender, N.A. & Moore, J.H. Moore Automating biomedical data science through tree-based pipeline optimization. In: *Applications of Evolutionary Computation* 123–137 (2016).

[48] R Core Team R: A language and environment for statistical computing. https://www.R-project.org/. (R Foundation for Statistical Computing, Vienna, 2018)

[49] Python Software Foundation. Python Language Reference, version 2.7. https://www.python.org

[50] ACOG Committee Opinion, number 755 Vol. 132, No. 4, October 2018 (Replaces Committee Opinion No. 534, August 2012)

[51] Manchanda R, Blyuss O, Gaba F, Gordeev VS, Jacobs C, Burnell M, Gan C, Taylor R, Turnbull C, Legood R (2018). Current detection rates and time-to-detection of all identifiable BRCA carriers in the Greater London population. J. Med. Genet..

[52] Beitsch PD, Whitworth PW, Hughes K, Patel R, Rosen B, Compagnoni G, Baron P, Simmons R, Smith LA, Grady I (2018). Underdiagnosis of hereditary breast cancer: Are genetic testing guidelines a tool or an obstacle?. J. Clin. Oncol..

[53] Zhou H, Jin Y, Dai L, Zhang M, Qiu Y, Tian J, Zheng J (2020). Differential diagnosis of benign and malignant thyroid nodules using deep learning radiomics of thyroid ultrasound images. Eur. J. Radiol..

[54] Guo Y, Hu Y, Qiao M, Wang Y, Yu J, Li J, Chang C (2018). Radiomics analysis on ultrasound for prediction of biologic behavior in breast invasive ductal carcinoma. Clin. Breast Cancer..

[55] Du Y, Fang Z, Jiao BJ, Xi G, Zhu C, Ren Y, Guo Y, Wang Y (2020). Application of ultrasound-based radiomics technology in fetal lung texture analysis in pregnancies complicated by gestational diabetes or pre-eclampsia. Ultrasound Obstet. Gynecol..

[56] Liu Z, Wang S, Di Dong JW, Fang C, Zhou X, Sun K, Li L, Li B, Wang M, Tian J (2019). The applications of radiomics in precision diagnosis and treatment of oncology: Opportunities and challenges. Theranostics..

