# Supplementary material for: Germline BRCA 1-2 status prediction through ovarian ultrasound images radiogenomics: a hypothesis generating study (PROBE study)
Source: Sci Rep. 2020 Oct 5;10:16511. doi: 10.1038/s41598-020-73505-2 (PMC7536234; doi:10.1038/s41598-020-73505-2)
Supplement: Supplementary file 1 — Supplementary Information. [file 41598_2020_73505_MOESM1_ESM.docx]

**GERMLINE BRCA 1-2 STATUS PREDICTION THROUGH OVARIAN ULTRASOUND IMAGES RADIOGENOMICS: A HYPOTHESIS GENERATING STUDY (PROBE STUDY)**

Camilla Nero^1^, Francesca Ciccarone^1^, Luca Boldrini^2^, Jacopo Lenkowicz^2^, Ida Paris^1^, Ettore Domenico Capoluongo^3^, Antonia Carla Testa^1^, Anna Fagotti^1^, Vincenzo Valentini^2^, Giovanni Scambia^1^

^1^ Fondazione Policlinico Universitario A. Gemelli IRCCS, Gynecologic Oncology, Dipartimento per le Scienze della salute della donna, del bambino e di sanità pubblica, Roma, Italia

^2^ Fondazione Policlinico Universitario A. Gemelli IRCCS, Dipartimento di Diagnostica per immagini, radioterapia oncologica ed ematologia, Roma, Italia

^3^ Department of Molecular Medicine and Medical Biotechnology, Federico II University - CEINGE, Advanced Biotechnology, Naples

*Table S1. Number of overlapping features (all) among US machines*

| *US machines* | *Overlapping features* |
| --- | --- |
| *Voluson – Esaote* | *14* |
| *Voluson – Toshiba* | *20* |
| *Voluson – Samsung* | *22* |
| *Esaote – Samsung* | *20* |
| *Esaote – Toshiba* | *22* |
| *Samsung – Toshiba* | *18* |

*Table S2. Number of overlapping features (statistically significant) among US machines*

| *US machines* | *Overlapping features* |
| --- | --- |
| *Voluson – Esaote* | *0* |
| *Voluson – Toshiba* | *0* |
| *Voluson – Samsung* | *2* |
| *Esaote – Samsung* | *0* |
| *Esaote – Toshiba* | *0* |
| *Samsung – Toshiba* | *0* |

*Supplementary Table 3.* *Different machine learning models performance for the different datasets*

| Eco type | Model | Acc. train | Acc. test | Spec. test | Sens. test | NPV test | PPV test | Kappa test |
| --- | --- | --- | --- | --- | --- | --- | --- | --- |
| All | Logit | 0.620 | 0.542 | 0.479 | 0.666 | 0.739 | 0.393 | 0.125 |
| Voluson | Logit | 0.740 | 0.625 | 0.587 | 0.720 | 0.840 | 0.409 | 0.250 |
| Toshiba | Logit | 0.710 | 0.659 | 0.760 | 0.545 | 0.655 | 0.666 | 0.308 |
| Samsung | Logit | 0.763 | 0.589 | 0.906 | 0.166 | 0.591 | 0.571 | 0.080 |
| Esaote | Logit | 0.870 | 0.735 | 0.800 | 0.642 | 0.761 | 0.692 | 0.447 |
| All | SVM | 0.806 | 0.609 | 0.540 | 0.746 | 0.808 | 0.451 | 0.247 |
| Voluson | SVM | 0.855 | 0.715 | 0.777 | 0.560 | 0.816 | 0.500 | 0.326 |
| Toshiba | SVM | 0.905 | 0.595 | 0.720 | 0.454 | 0.600 | 0.588 | 0.177 |
| Samsung | SVM | 0.848 | 0.482 | 0.187 | 0.875 | 0.666 | 0.446 | 0.055 |
| Esaote | SVM | 0.900 | 0.705 | 0.550 | 0.928 | 0.916 | 0.590 | 0.440 |
| All | XGB | 0.899 | 0.630 | 0.812 | 0.333 | 0.665 | 0.520 | 0.148 |
| Voluson | XGB | 0.950 | 0.645 | 0.816 | 0.298 | 0.702 | 0.446 | 0.157 |
| Toshiba | XGB | 0.681 | 0.587 | 0.840 | 0.230 | 0.606 | 0.505 | 0.162 |
| Samsung | XGB | 0.860 | 0.550 | 0.690 | 0.379 | 0.574 | 0.502 | 0.100 |
| Esaote | XGB | 0.930 | 0.573 | 0.819 | 0.130 | 0.675 | 0.264 | 0.102 |
| All | autoML | 0.680 | 0.639 | 0.912 | 0.179 | 0.652 | 0.546 | 0.107 |
| Voluson | autoML | 0.797 | 0.716 | 0.865 | 0.407 | 0.733 | 0.617 | 0.307 |
| Toshiba | autoML | 0.761 | 0.632 | 0.804 | 0.372 | 0.660 | 0.557 | 0.194 |
| Samsung | autoML | 0.716 | 0.586 | 0.677 | 0.468 | 0.623 | 0.528 | 0.151 |
| Esaote | autoML | 0.820 | 0.670 | 0.895 | 0.208 | 0.700 | 0.492 | 0.121 |
